# Highly sensitive and multiplexed one-step RT-qPCR for profiling genes involved in the circadian rhythm using microparticles

**DOI:** 10.1038/s41598-021-85728-y

**Published:** 2021-03-19

**Authors:** Mi Yeon Kim, Seungwon Jung, Junsun Kim, Heon Jeong Lee, Seunghwa Jeong, Sang Jun Sim, Sang Kyung Kim

**Affiliations:** 1 Center for Molecular Recognition Research, Materials and Life Science Research Division, Korea Institute of Science and Technology(KIST), Seoul, KS013 Korea; 2 Department of Chemical Biological Engineering, Korea University, Seoul, KS013 Korea; 3 Department of Psychiatry and Chronobiology Institute, Korea University College of Medicine, Seoul, KS013 Korea

**Keywords:** Genetics, Molecular biology, Biomarkers, Diseases, Health care

## Abstract

Given the growing interest in molecular diagnosis, highly extensive and selective detection of genetic targets from a very limited amount of samples is in high demand. We demonstrated the highly sensitive and multiplexed one-step RT-qPCR platform for RNA analysis using microparticles as individual reactors. Those particles are equipped with a controlled release system of thermo-responsive materials, and are able to capture RNA targets inside. The particle-based assay can successfully quantify multiple target RNAs from only 200 pg of total RNA. The assay can also quantify target RNAs from a single cell with the aid of a pre-concentration process. We carried out 8-plex one-step RT-qPCR using tens of microparticles, which allowed extensive mRNA profiling. The circadian cycles were shown by the multiplex one-step RT-qPCR in human cell and human hair follicles. Reliable 24-plex one-step RT-qPCR was developed using a single operation in a PCR chip without any loss of performance (i.e., selectivity and sensitivity), even from a single hair. Many other disease-related transcripts can be monitored using this versatile platform. It can also be used non–invasively for samples obtained in clinics.

## Introduction

Quantitative reverse transcription polymerase chain reaction (RT-qPCR) is the gold standard technique for analyzing the mRNA of a specific gene^1, 2^. It is widely used in clinics for infectious diseases^3, 4^ and chronic diseases such as cancer^5, 6^ and metabolic syndromes^7^. Most chronic diseases are difficult to define because they are often characterized by extensive genetic markers composed of tens of mRNAs and miRNAs^8^. Sequencing technology has allowed the identification of various gene markers responsible for a single phenotype. Therefore, recent molecular diagnosis is now performed using a panel of biomarkers, which typically includes several to tens of different mRNAs^9^. For example, patients with mood disorders may have altered transcripts that are associated with disruptions in biological rhythms^10^. Many studies have estimated circadian dysfunction in patients using mRNA profiling of circadian genes^11, 12^. These disease-associated patterns have been validated through comparison of patients’ circadian rhythms to those of normal people over time^13^.

Although RT-qPCR is the most suitable assay to precisely quantify RNA targets, it is a laborious process to screen several genes at the same time. The limited quantity of sample in clinics makes the detection of multiple RNAs even more difficult^14^. The general approach to analyze multiple genes in a minimal sample is to reverse transcribe RNA using poly T primers or random hexamers. Next, the each target is separately amplified. Although this process allows analyzing a number of the genes from a single sample, it also involves complicated hands-on operations (such as splitting the samples and filling different reagents into each reaction). Therefore, it may suffer from contamination issues^15^. Another weakness of this classic approach is the non-target signal, because universal RT primers (such as poly T or hexamers) propagate irrelevant products besides target cDNA^16^.

A preamplification process may enhance the sensitivity of real-time RT-PCR, especially for low abundance genes. This process ensures higher cDNA amounts prior to splitting it, which can then expand the number of analyzable target genes^17^. Targeted preamplification is usually conducted using multiplex PCR, which is highly complex reaction that is subject to interference from the mixed amplification of many targets. Therefore, the annealing time is extended to > 3 min to maintain sufficient efficiency in preamplification^18^. Furthermore, the effects of using several PCR additives, which may improve enzymatic reactions must be optimized^19^.

We previously reported the original primer-incorporated networks (oPINs) that enabled efficient enzymatic reactions of RT and PCR in a microparticle^20^. Microparticle based RT-qPCR demonstrated high sensitivity and selectivity for target RNAs of high complexity including cellular total RNA^21^. An upgraded version of this assay was later developed for multiplex analysis through the integration of thermo-responsive carriers in oPIN. The thermo-responsive PIN (tPIN) simultaneously can perform multiplex RT-qPCR by assembling the microparticles into one chamber because it stores the specific primers in each microparticle^28^.

In this study, we demonstrate the clinical utility of the highly sensitive, selective, and multiplexed one-step RT-qPCR platform using tPIN. Additionally, the particle-based multiplex method is more cost-effective than the conventional method. For multiple analysis of 8 targets, one can finish the experiment with the same amount of resources as conventional single assay. Using this platform, the expression of nine different circadian markers(PER2, PER3, CLOCK, ARNTL, NR1D1, NR1D2, CRY1, CRY2, and NPAS2) and one reference marker were monitored from human hair follicles and synchronized cells every 4 h for 24 h. The pre-concentration of RNAs (that was achieved in the tPINs) increased the sensitivity of this platform tenfold. Based on the improved sensitivity, we successfully quantified mRNAs of eight circadian genes from < 200 pg of total RNA and also from a single hair follicle. This microparticle-based one-step RT-qPCR platform can be also used in other clinical applications to profile extensive transcripts from a limited amount of sample.

## Material and methods

### Primer design

Primers were synthesized and purified using PAGE purification (IDT, USA). For the detection of circadian gene expression levels, forward and reverse primers were designed as shown in Table 1. In order to crosslink the primers of the PIN particles and purify the target gene, the acrydite and EcoRI sites were added at the 5′ terminus of the reverse primer.

**Table 1:** List of target specific primer sequences for real time RT-qPCR analysis used in this circadian study.

Target gene	Length (bp)	Forward primer (5′ → 3′)	Reverse Primer (5′ → 3′)
PER2	108	GTATCCATTCATGCTGGGCT	TCGTTTGAACTGCGGTGAC
PER3	146	TCAGTGTTTGGTGGAAGGAA	TCTGGGTCAGCAGCTCTACA
CLOCK	137	AGTGGATTTGGCTTCAGACT	TTCAATGCCAAGTTCTCGTC
ARNTL	123	TGTGCTAAGGATGGCTGTTC	GCCCTGAGAATGAGGTGTTT
CRY1	102	CCACGAATCACAAACAGACG	CTCCAATGTGGGCATCAAC
CRY2	145	TGCAGGTTGTACTCTGCTGC	TGAAGAACTCAGCAAACGGG
NPAS2	136	GCAAGGTCTTGAAAGGGTGT	TCTTCTCAGAGGCAGCTTGA
NR1D1	128	GAAGCTGCCATTGGAGTTGT	AAGACATGACGACCCTGGAC
NR2D2	148	GCCTCCACAGAGTTGACGTT	GAGCAGGGGATCTGCTAAAC
PPIA	124	TCCTTTCTCTCCAGTGCTCAG	CACCGTGTTCTTCGACATTG

### Synthetic RNA & synthetic DNA

Specific sequences (CCCTATAGTGAGTCGTATTA) were added at the 3′ terminus of the synthetic DNA, which was synthesized with high purity using PAGE purification (IDT, USA). To construct synthetic RNA, the T7 promoter (5′-TAATACGACTCACTATAGGG-3′) and synthetic DNA of each gene were mixed in the same amount of 1 ug/ul and incubated overnight at 37° C using HiScribe T7 High Yield RNA Synthesis kits (New England Biolabs, USA) for IVT(in vitro transcription). We synthetized 2ug/ul of synthetic RNA similar to the actual mRNA using reverse transcription from synthetic DNA.

### Preparation of encoded tPIN particles

The pre-polymer solution for tPIN particle preparation included a mixture of the following: 20% v/v Poly(ethylene glycol) diacrylate (PEG-DA, Sigma-Aldrich, Mn = 700), 40% v/v Poly(ethylene glycol) (PEG, Sigma-Aldrich, Mn = 600), 35% v/v nanoparticles with 2% low melting agarose and 200uM free DNA as a reverse primer and 5% v/v Darocur 1173 (Sigma-Aldrich). The free reverse primer was captured in 2% low melting agarose. The free reverse primer dose not react in RT process because the nanocapsules inhibit RT non-specific binding product^28^. The solution for the tPIN was finalized by mixing the pre-polymer solution and 1 mM acrydited DNA as a forward primer with a volume ratio of 9:1. The tPINs were produced by dropping the pre-polymer solution on the PDMS, which was pre-patterned with eight different dot codes using a jetting system (Arrayer 2000, Advanced Technology Inc., Korea)^20^. (Figure S1) The solution then underwent 10 sec of UV exposure at 4.5 mJ/cm2. The preparation of the tPINs was completed through a rinsing process using 1 × PBS buffer of 0.05% Tween-20 to remove the porogens and the unbound primers. The completed particles were stored at room temperature soaked in a 1 × PBS buffer with 0.05% Tween-20.

### Cell culture

Cells from the HeLa human cervical cancer cell line were cultured in Minimum Essential Media (MEM; Gibco) supplemented with 10% fetal bovine serum (FBS; Gibco), 50 U/ml penicillin and 0.05 mg/ml streptomycin at 37 °C with 5% CO2. For the experiments, 5*10^6 cells were plated on T25 flask and cultured in MEM contacting 5% FBS for 3–4 days. The cells reached confluence after approximately three days under these conditions.

### 10uM Forskolin treatment

For the cell cycle synchronization, the medium was changed to MEM supplemented with Forskolin at 0 h (10uM in final concentration, Sigma-Aldrich). At 2 h, these media were replaced with serum-free MEM supplemented with 50U/ml penicillin–streptomycin. At the indicated time, the cultured cells were washed three times with ice-cold PBS and harvested in 1 ml TRIzol reagent (Thermofisher). The samples were frozen and stored at − 80 °C until the extraction of whole cell RNA^25, 26^.

### RNA extraction

#### Total RNA from HeLa cell

HeLa cells in T25 flaks were centrifuged at 1,000 rpm for 3 min. The pellet was homogenized in 1 ml Trizol (Invitrogen) every 4 h for 48 h, and stored at -80° C before extraction of total RNA. The total RNA was extracted from the pellet after 48 h according to the manufacturer’s instructions.

#### mRNA from human hair follicle cells

**Figure 1:**
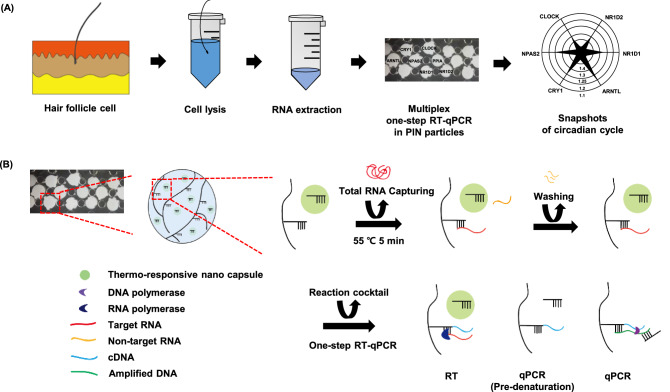
Principle of one step RT-qPCR process in primer immobilized network(PIN) microparticles. (**A**) Schematic representation of circadian snapshots for multiplex detection in one step RT qPCR process from human hair follicle Gene expression level was demonstrated using the C_t_ values of the PIN particles. (**B**) Process of RNA capturing using microparticles possessing forward and reverse primer was demonstrated at 55 ºC for 5 min. After the washing process, the one step RT-qPCR cocktail mix was introduced to perform one step RT-qPCR process The qPCR primer was embedded in the nanocapsule (a thermo responsive polymer represented in green, tPIN to prevent them to participate in the RT (reverse transcription) process In the qPCR pre denaturation process, the nanocapsule was melted at 95 ºC where the qPCR primer is free to move thus, enabling the qPCR reaction.

We plucked five scalp hairs from normal humans at 4 h intervals around 24 h. The hair, including the hair follicle cells, were immediately placed in 700 ul of RNA later solution and stored at -80 °C. After the overnight incubation, the solution was dissolved and the total mRNA was extracted using an RNeasy micro kit (Qiagen, USA). DNA contamination is removed by on-column DNase digestion. 20 ng of the RNA carrier was added to the homogenization process in order to extract RNA from a small amount of cells. Every study participant was informed in detail about the purpose and procedures of the study prior to signing an informed consent form. The study protocol was approved by the Institutional Review Board of Korea University’s Anam Hospital and was conducted in accordance with the Declaration of Helsinki. The quality and quantity of RNA after purification were examined using a Nanodrop (Synergy Mx biotek), and compared to examples of pure RNA results found in the Nanodrop Expert User’s Guide. The amount of total RNA from each sample was approximately 90–150 ng/μl. (Fig. 1A).

### Real-time one-step RT-quantitative PCR with PIN

Micro particle-based one-step RT-qPCRs were conducted using UltraFast LabChip Real-time PCR G2-4 (Nanobiosys, Korea). Five PIN particles for one target were assembled into one PCR chamber, and 1ul template RNA was captured at 55 °C for 5 min. After RNA removal, the PIN-based one-step RT-qPCR mastermix was made for a final volume of 10ul. This volume was enough to fill the PCR chamber. 1ul deionized water was mixed with the following: 5ul 2 × SYBR Green mastermix (Nanobiosys, Korea), 2ul 5X himebio SYBR Green mastermix (Himebiotech, Korea), 1ul 1X RTase (Thermo scientific, USA), 1ul 1X Ribolock (Thermo Scientific, USA) except forward primer, reverse primer, and template RNA. After reverse transcription was performed for 5 min at 42 °C, qPCR was performed. A two-step amplification program was used, with the following parameters: 3 s at 95 °C (denaturation) and 30 s at 60 °C (annealing) for 50 cycles after the pre-denaturation step at 95 °C for 20 s. The process only requires 39 min. The fluorescent images were saved at each cycle. Their intensities were recorded and all of the data were normalized by the maximum intensities. (Fig. 1B).

### Single cell detection

Each of the hydrogel particles, with a maximum removal of 0.05% PBST buffer, was placed on the glass chip that was divided into the PDMS mold. One to five HeLa cells were dropped on each hydrogel particle respectively using CellenONE (Cellenion, France). 10 nl RIPA lysis buffer was directly dropped onto the particles and incubated at 4 °C for 10 min so that the cells could be lysed. After incubation at 55 °C for 5 min, the 10ul RT reagents were mixed with 1ul 1X RTase, 1ul 1X Ribolock, 2ul 10 mM dNTP, and 5ul 5X reaction buffer for Reverse Transcription. This step was performed to specifically induce the target RNA that was hybridized using the particle primers. After Reverse Transcription was performed for 30 min at 42 °C, the cDNA-synthesized particles were assembled in an UltraFast LabChip and filled into a10ul PCR cocktail mixture. This mixture was made of 5ul 2X Rapi SYBR green Mx (Genesystem, Korea), 1ul 40X SYBR green (Genesystem, Korea), 4ul deionized water. PCR was performed on UltraFast LabChip Real-time PCR G2-3 (Nanobiosys), which included 4 sec at 95 °C (denaturation) and 10 sec at 60 °C (annealing & extension) after the pre-denaturation step at 95 °C for 8 sec in Pre-denaturation. (Figure S2A).

## Results

### Validation of one-step RT-qPCR for mRNA quantification

#### Real-time PCR performance of thermo-responsive PIN (tPIN)

**Figure 2:**
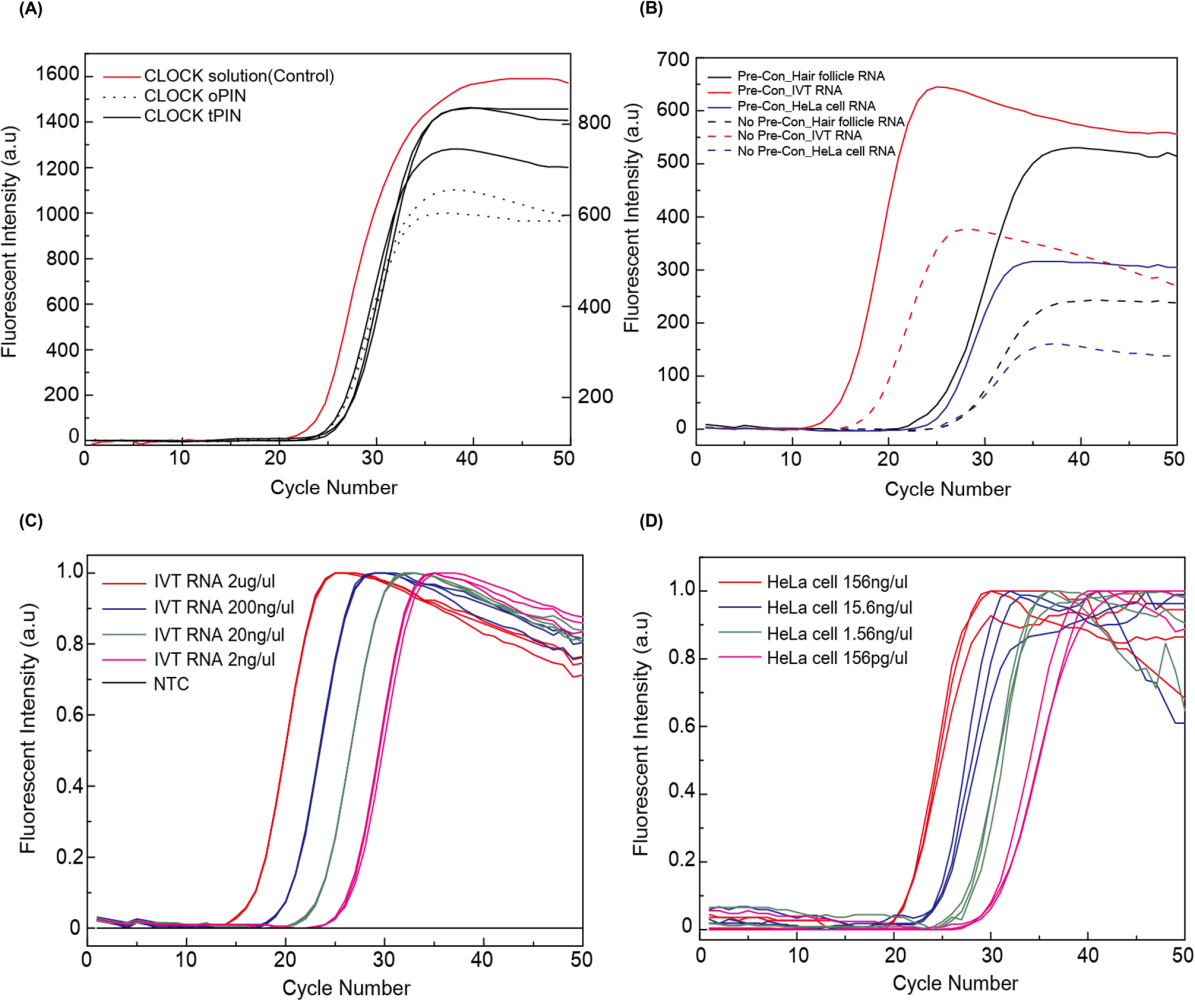
Validation of PIN(Primer-Immobilized Network) based PCR. (**A**) Comparison between the conventional solution and PIN (oPINand tPIN) based PCR. (**B**) Effect of pre-concentration (Pre-Con) step before incubation of PIN particles with one-step RT-qPCR mix from different RNA origins. In case of pre-concentration, the Ctvalues appeared early (~ 3) and enhanced fluorescent intensity was observed compared to non pre-concentrated ones. In case of HeLa cell RNA and hair follicle RNA, it was confirmed that this assay had a tenfold high sensitivity. (**C**) Concentration dependent one-step RT-qPCR graphs of the IVT RNA templates. Difference in Ct values were uniform (~ 3.3) corresponding to the ten-fold difference in template concentration. Interestingly, PIN based one-step RT-qPCRshowed no signals for NTC (No template control) until 50thcycle. (**D**) Limit of detection (LOD) test for HeLa cells using different concentration of total RNA.

One-step RT-qPCR requires specific RT and PCR primers. This is also true for our microparticle-based RT qPCR. However, when both primers are fixed on the substrate in the solid-phase reaction, the efficiency of the enzymatic reaction is significantly decreased^20–22^. The tPIN particles have great advantages over this inefficiency. tPIN particles contain nanocapsules with thermo-responsive materials that only release PCR primer during the PCR cycles in which the reaction occurs at high temperatures. As a primary step, we examined the efficiency of the PCR amplification using tPIN particles of high porosity for mRNA delivery. In Fig. 2A, CLOCK, one of representative circadian genes, was selected as the first target. PIN qPCR and solution qPCR were performed using synthetic cDNA. According to the identical characteristics of the amplification in the original PIN (oPIN) and the tPIN, the thermo-responsive carriers supply the primers well. The C_t_ value of the particle qPCR was delayed by 2.37 with standard deviation of 0.35 compared to that of the conventional solution qPCR. C_t_ value of the particle qPCR (oPIN and tPIN) were similar. The standard deviation in C_t_ value is 0.35, which reflects the stability of particle qPCR. The C_t_ value of the particle qPCR is slightly bigger than that of the conventional solution because of the lower degree of primer freedom in the PIN matrix.

The circadian rhythm in humans is a 24-h cycle that is involved in multiple processes, including endocrine secretions, metabolic processes, neuronal activities, immune functions, and sleep. These processes can become disturbed, and contribute to disease (such as mental disease), if the circadian rhythm is disturbed. The circadian rhythm can be evaluated using related transcripts. The PCR assays of previous mentioned genes were set up. We validated their performance in comparison with the assays using oPIN particles. With regard to these eight circadian genes, the tPIN qPCRs had no recognizable difference from the oPIN’s in C_t_ values. However, the signal in tPIN qPCR was slightly more intense than that it was in oPIN qPCR. In single-plex qPCR, the two modes of PIN qPCR seemed to be equal. (Figure S3).

#### One-step RT-qPCR validation

In addition to validating tPIN qPCR with DNA templates, the efficiency of one-step RT-qPCR was evaluated using synthetic RNA. First, CLOCK RNA was prepared through in vitro transcription of synthetic cDNA. The most efficient RT condition was determined at 42 °C and 5 min by varying the reaction time and temperature (Figure S4). Every RT in this study was performed under these conditions. The efficiency of one-step RT-qPCR was calculated as 98% by serial dilution of CLOCK IVT RNA, as shown in (Fig. 2C). In one-step RT-qPCR, the two enzyme reactions are connected within 30 min. In contrast to the conventional RT-qPCR, false signal has not been observed in the no template control (NTC). One prior study described that the risk of NTC signal decrease due to random binding when a fixed primer is excluded in the setting of one-step RT-qPCR. This improvement is explained by the fact that fixed primers require a higher affinity (than does a freely suspended primer) in order to bind free templates^21^. In addition, non-specific binding between primers and RNAs can also be inhibited under the tPIN environment, which isolates the PCR primers during RT^28^. The fluorescent signal of qPCR is now confirmed as a positive signal from the correct response to the target RNA. The amplicons from the one step assay was confirmed using gel electrophoresis (Figure S1). In Figure S6, the CLOCK targeted oPIN and tPIN based RT-qPCR showed similar C_t_ values. The C_t_ value of the tPIN based RT-qPCR for PTC increased by 1.0 compared to that of the conventional RT-qPCR, with standard deviation of 0.15. It is worth mentioning that PIN based RT-qPCR became even closer to the conventional liquid phase RT-qPCR when it was compared with PCR. This comparison also confirmed that the intensity of the particles’ fluorescence signals was very uniform.

#### The effects of RNA pre-concentration in PIN particles

In order to be able to detect mRNAs with high precision, even in a small amount, it is key to optimize the sensitivity of the test. One of the key advantages of particle-based RT-qPCR is that the target RNA is pre-concentrated, because the RT primers are covalently immobilized in the particle matrix to bind with the target RNAs. Incubation of the RNA samples in the particles can increase the actual concentration of the sample. The effect of pre-concentration can also deliver more target RNAs to the primers. We performed pre-concentration, and optimized the study conditions by varying the time and temperature. The longer the time, the earlier the amplification signal. The signal steadily improved for up to 5 min, after which the beneficial effect of pre-concentration approached saturation. (Figure S7A) Therefore, we chose five minutes of pre-concentration to run the process most efficiently. The effect of pre-concentration on temperature was also confirmed. The most effective temperature (55 °C) was chosen as the pre-concentration temperature. (Figure S7B) 1ul RNA samples were pre-concentrated to the PIN particles at 55 °C for 5 min. The C_t_ value was shifted down by approximately three cycles when compared with the reactions performed without pre-concentration (Fig. 2B). This is theoretically consistent with a tenfold higher concentration expected in this protocol, because the RNA concentration is diluted by 1/10 in the mastermix reagents from the conventional protocols. Since the RNA goes into the PIN particle and remains there during delivery of the mastermix, the assay may be protected from dilution (which is inevitable using conventional assays). This improved sensitivity was the same for complex RNA samples, such as total RNA from HeLa cells and hair follicles. (Fig. 2B) Therefore, every experiment in this study was performed using a 5-min pre-concentration process.

#### Particle-based one-step RT-qPCR efficiency and LOD (Limit of Detection) from total cell RNA

The finalized protocol of the one step RT-qPCR was validated using pure synthetic RNA and total RNA from HeLa cells. (Fig. 2C andD) The efficiency of the particle based one-step assay was measured in triplicate experiments using serial diluted standards of pure RNA, and with the total RNA. The amplification efficiencies of the both cases were > 99%. This high efficiency with a complex RNA sample represents the reliable performance of this assay in selectivity, which is a key element for practical applications. We performed gel electrophoresis to confirm that the final product of amplification was the target mRNA. (Figure S5) In addition to the reliability, the limit of detection (LOD) is also critical to clinical utility. 156 ng/µl RNA was extracted from 6175 cells whereas the 156 pg/µl RNA was extracted from 6 cells. The efficiency of one-step RT-qPCR was ~ 98% for both IVT and HeLa cell RNA. A small amount (156 pg) of total RNA was enough to detect the target mRNA with a C_t_ value of 29 (Fig. 2D). The amount of RNA in a single cell ranges 10-30 pg^28^. Several cells, therefore, may be enough to achieve reliable mRNA quantification using this novel RT-qPCR method.

### mRNA expression of circadian rhythm genes from HeLa cells and human hair follicle cells

#### Multiplexed assay

##### Multiplex one-step RT-qPCR for circadian genes

**Figure 3:**
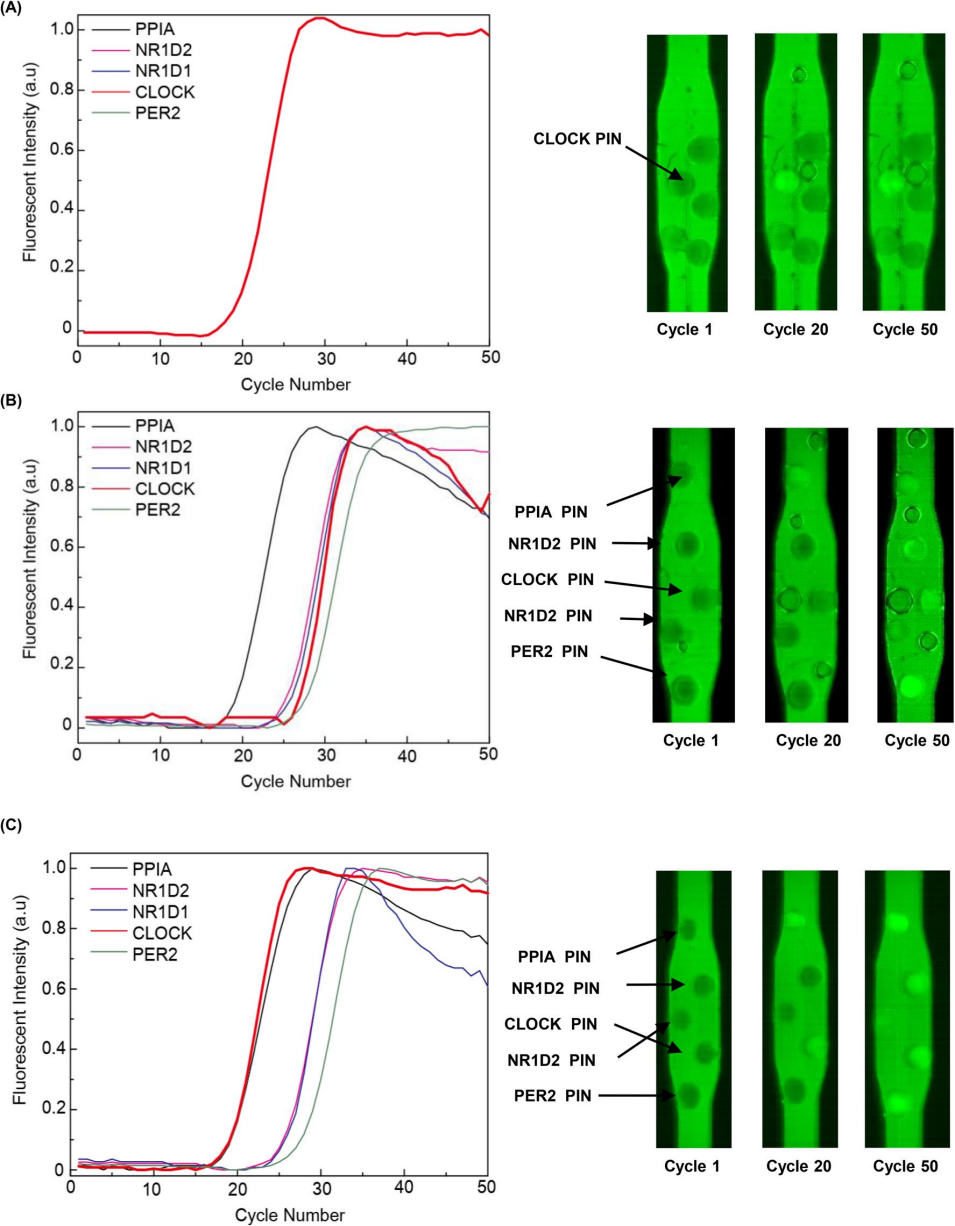
Multiplex spike in one step RT-qPCR assay for various genes and their corresponding fluorescence images. (**A**) Spike in RT-qPCR strategy for CLOCK IVT RNA in which the microparticle targeting only the CLOCK gene was brightened. (**B**) Spike-in RT-qPCR process for HeLa total cell RNA. (**C**) Spike in approach for CLOCK IVT RNA in HeLa total cell RNA CLOCK particle proceeded without interruption with an early C_t_ value of 19.1 from 26.9 Fluorescence signals of the multiplex qPCR with five different PIN particles showed specific fluorescence signals for the targets.

Multiplexed RT qPCR is appropriate to screen RNA targets, given its efficient use of high throughput sequencing, which is well suited for biomarker discovery. A multiplex assay with tPIN is achieved simply by adding more particles that are targeting RNA. As mentioned above, the microparticles storing each specific primer behave as separate reactors. Therefore, there is no cross-talk between these microparticles, which is the most serious problem with conventional multiplex PCR. In order to demonstrate the independent nature of the reactions in a multiplexed assay, a five-plex assay was performed using five targeting microparticles with samples of total RNA and spiked-in synthetic RNA. As shown in Fig. 3, the particles targeted that PER2, CLOCK, NR1D1, NR1D2, and PPIA were assembled in a PCR chamber to conduct multiplex one-step RT-qPCR. First, a sample of 1 μl containing CLOCK synthetic RNA was analyzed in a multiplexed configuration to evaluate its specificity. As expected, CLOCK was the only gene to produce a nice amplification curve, while the others stay completely dark (Fig. 3A). This result suggests that the enzymatic reactions were independently performed in each particle without crosstalk between the particles. Next, a multiplex assay of circadian genes was performed using total RNA from the cell lysates. As shown in Fig. 3B, five different particles showed individual signals resulting in their respective C_t_ values (PER2 Ct : 27.7 , CLOCK Ct : 26.9, NR1D1 Ct : 25.9, NR1D2 Ct : 25.3, PPIA Ct: 19.3). When CLOCK synthetic RNA was added into the total RNA (Fig. 3C), the C_t_ values of the four particles (except CLOCK) were not affected except CLOCK (Fig. 3C). We were able to confirm the individual RT-qPCR of each particle, as well as the reproducible quantifications, because the variation of C_t_ values in Fig. 3A and C reside within 0.2. The C_t_ value of the CLOCK gene was 19.1, which is very close to the C_t_ value (19.3) that was obtained when only synthetic RNA was analyzed. This value was obtained without interruption from many other RNAs in the total RNA. These multiplex assays confirm that tPIN one-step RT-qPCR can be easily multiplexed with excellent particle independence and high specificity for the individual targets.

##### Circadian gene expression of synchronized HeLa cells

**Figure 4:**
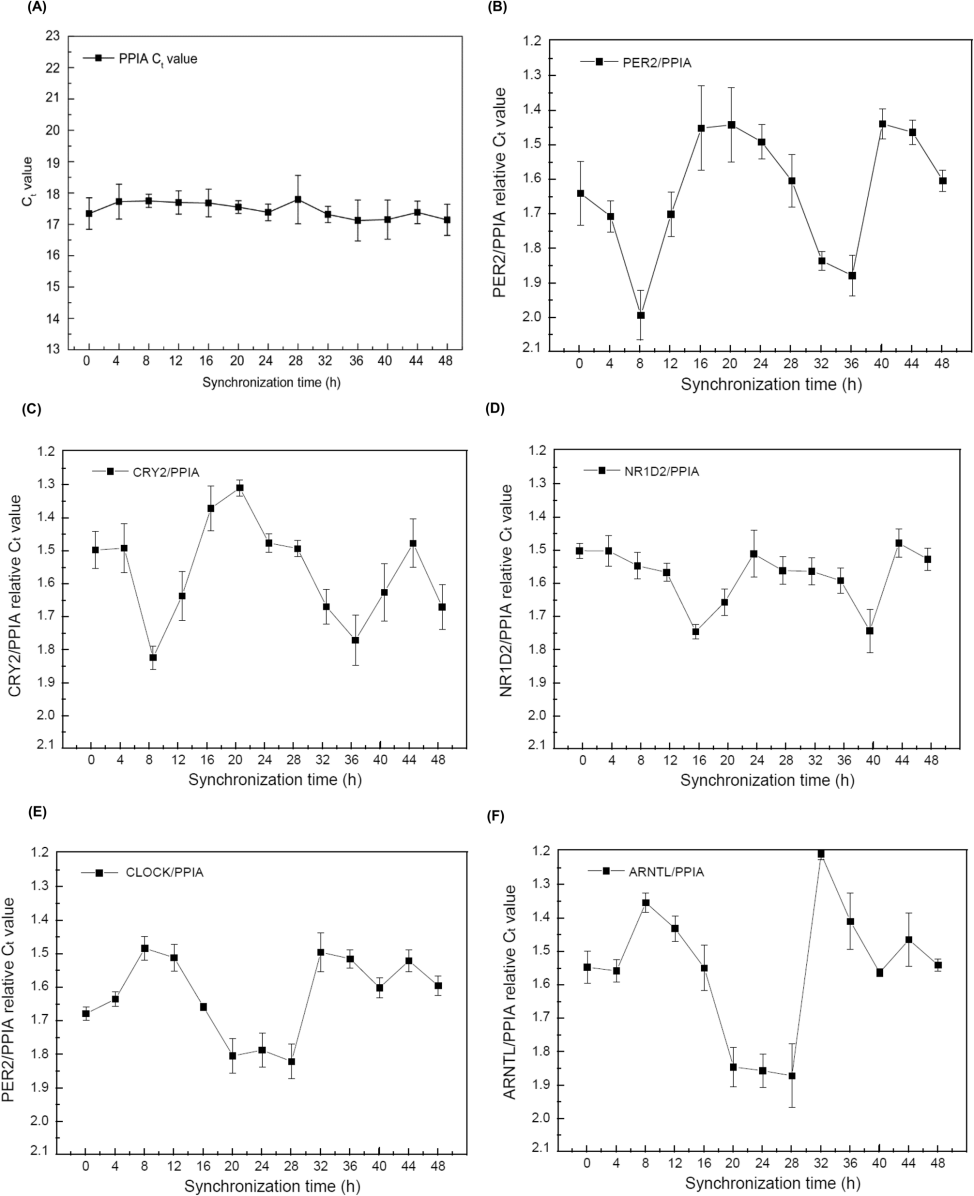
Synchronization test for HeLa cells with forskolin induced circadian gene expression of PER 2 CRY 2 CLOCK, ARNTL, NR 1 D 2 and PPIA mRNAs. (**A**) Expression level of internal control gene (PPIA) for 48 h with a time interval of 4 h (n = 5) was analysed. (**B**–**F**) Relative C_t_ values of circadian rhythm genes with respect to the internal control (n = 5). PER2, CRY2, and CLOCK, ARNTL showed an inverse fluctuation during the whole 48 h. Cyclic fluctuation occurred at 8 h in NR1D2 when compared to that of PER2, CRY2.

We examined cells’ circadian cycle using the multiplexed analysis with total RNA from the synchronized human cell line. As shown in Fig. 4, we analyzed the RNA expression levels of each circadian gene in the total RNA of HeLa cells every 4 h for 48 h. The particles targeting six different genes were assembled in a PCR chamber, and the multiplex assay was performed for samples of total RNA at different time points. The housekeeping gene PPIA was used as a reference gene. The C_t_ value of PPIA showed no significant fluctuation around a C_t_ value of 17 (p < 0.01) over all time points. The C_t_ values for every other gene were normalized to that of PPIA in order to compensate for any quantitative variations in the preparation of total RNA. In contrast to PPIA, PER2, and CRY2 showed distinct periodic fluctuations their expression level. The waves of CLOCK and ARNTL were in the opposite phase to those of PER2 and CRY2. In addition, NR1D2 had a delay of approximately 8 h compared to PER2 and CRY2. The cyclic fluctuations of these markers, and particularly the opposite wave forms between the two groups of genes, were consistent with findings in previous studies^26, 27^. Therefore, the multiplex one-step RT-qPCR can reflect the 24 h-periodic cycle of circadian rhythm RNA markers in cell line as good as conventional assays do (in which each target mRNA is measured separately).

#### The effects of RNA pre-concentration in PIN particles

##### Circadian gene expression in human hair follicles

**Figure 5:**
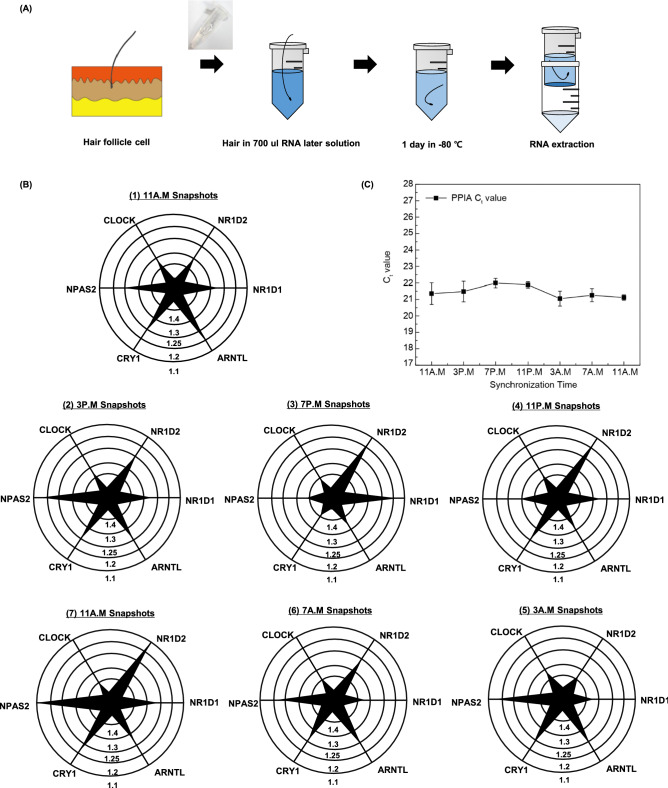
Circadian rhythm observed in 5 hair follicles for a synchronization time of 24 h. (**A**) RNA extraction procedure from the single individual with five different hair follicles Using total RNA collected from five different scalp hairs, real time one step RT-qPCR was performed to measure the expression of the genes. (**B**) Time course of expression profiles for each circadian gene for every 4 h starting from 11 A.M. for a total period of 24 h (n = 5) in radial graph. (**C**) Expression level of internal control (PPIA), for 24 h.

The circadian rhythm of a human was measured through the established multiplex assay. In addition to measuring the circadian cycle in multiple cellular samples, such as oral mucosa or white blood cells, recent studies have also quantified circadian gene expression in total RNA from human hair follicles^23^. Hair follicles can be obtained non-invasively, and they tend to be less vulnerable to degradation by RNase than is other human DNA. In order to measure the circadian rhythms of multiple genes, more than five hair follicles were plucked from a healthy human every 4 h in 24 h. The hair follicles were immediately placed in 700 ul of RNA later solution and stored at − 80 °C. (Fig. 5A) The multiplex one-step RT-qPCR was performed with 7 different classes of tPINs after extraction of RNAs from hair follicles. As in the previous experiments using cell lines, the gene of internal control, PPIA, showed a constant C_t_ value of 21 without significant change over time (*p* < 0.01). (Fig. 5C) In contrast, the circadian genes CLOCK, ARNTL, CRY1, NPAS2, NR1D1, and NR1D2 exhibited cyclic fluctuations. Their expression patterns are similar to the those of the synchronized cell line. NR1D2 had the highest expression in the morning from 7 to 11 am, while CLOCK was relatively more expressed around 7 pm compared to the one in the morning. Interestingly, the gene expression profile recovered within 24 h, as was observed in the comparison of the first and seventh radial diagrams in Fig. 5B. We identified all 10 genes present in Table 1, but displayed them except for genes that do not show much fluctuation over time. Because the profile of genes that sufficiently expressed in Hela cells and hair follicle cells are slightly different, the types of genes seen in manuscript are different (Table 1).

For validation purposes, the tPIN-based multiplexed one-step RT-qPCR was directly compared with conventional PCR. We divided a sample from the hair follicles evenly and measured the gene expression profiles among NPAS2, NR1D1, and PPIA. Conventional PCR for each gene was conducted, including RT for 1hour and qPCR for 1 h in the Light Cycler 480 real-time PCR system (Roche, USA). The gene expression profiles were very similar between conventional PCR and multiplex one-step RT-qPCR, although the absolute amplification of multiplex RT-qPCR was earlier than that of the conventional one by an approximate C_t_ of 2. (Figure S8) With regard to analytical performance of the RNAs, the quantitative PIN-based multiplex RT-qPCR was equivalent to the conventional method. However, the simplicity of this assay led us to also pursue tPIN-based multiplex analysis.

#### Multiplex quantitative analysis in more limited conditions

##### Single cell detection

As previously discussed, the amount of RNA contained in 6 cells is sufficient for a stable tPIN assay (Fig. 2D). If we reduce the reaction volume for RT and enhance the RNA concentration, we can quantify RNA from an even smaller sample size. For instance, RNA from a single cell can be theoretically measured using a decreased pre-concentration volume of 10-30 pg of RNA /10 nl of lysis buffer. In Figure S2A, we have minimized the reaction volume by dropping single cells into 360-440pL of media on a tPIN particle in the microwell. The input volume of the lysis buffer was limited to10 nl after pairing the cell and particle. For cell lysis and diffusion of those RNAs into the PIN, the particles were incubated in the well for 10 min. After this, the reagent for RT was added. After RT and qPCR in the particles, PER3 was amplified and quantified down to two cells. The CLOCK and ARNTL genes measured from a single cell had an approximate C_t_ value of 26. Considering that single mammalian cells usually have 10–30 pg of total RNA and the 10 nl of lysis reaction volume in this assay, the actual concentration of total RNA reacting with the particles may only be 1–3 ng/ul. This concentration is approximately 10 times larger than 156 pg, which was measured as an LOD of this platform with the general protocol using 1 ul of input RNA sample. Therefore, a difference of 3 in the C_t_ value means a difference of 10 times in the concentration. The C_t_ value of ~ 26 is quite reasonable (Figure S2B). In order to see whether the fluorescence signals from the particles were correct from the target amplification, gel electrophoresis was performed. On gel electrophoresis, the target sizes were confirmed with each single band at 146 bp for PER3, at 137 bp for CLOCK, and at 102 bp for ARNTL. Further customization of the assay protocol is necessary to obtain reliability, as the cell lysate was not purified. However, the tPIN demonstrated fair performance in RNA analysis from a single cell, and ensured its advantages in small sample analysis.

##### Snapshots of circadian cycle from a single hair follicle

**Figure 6:**
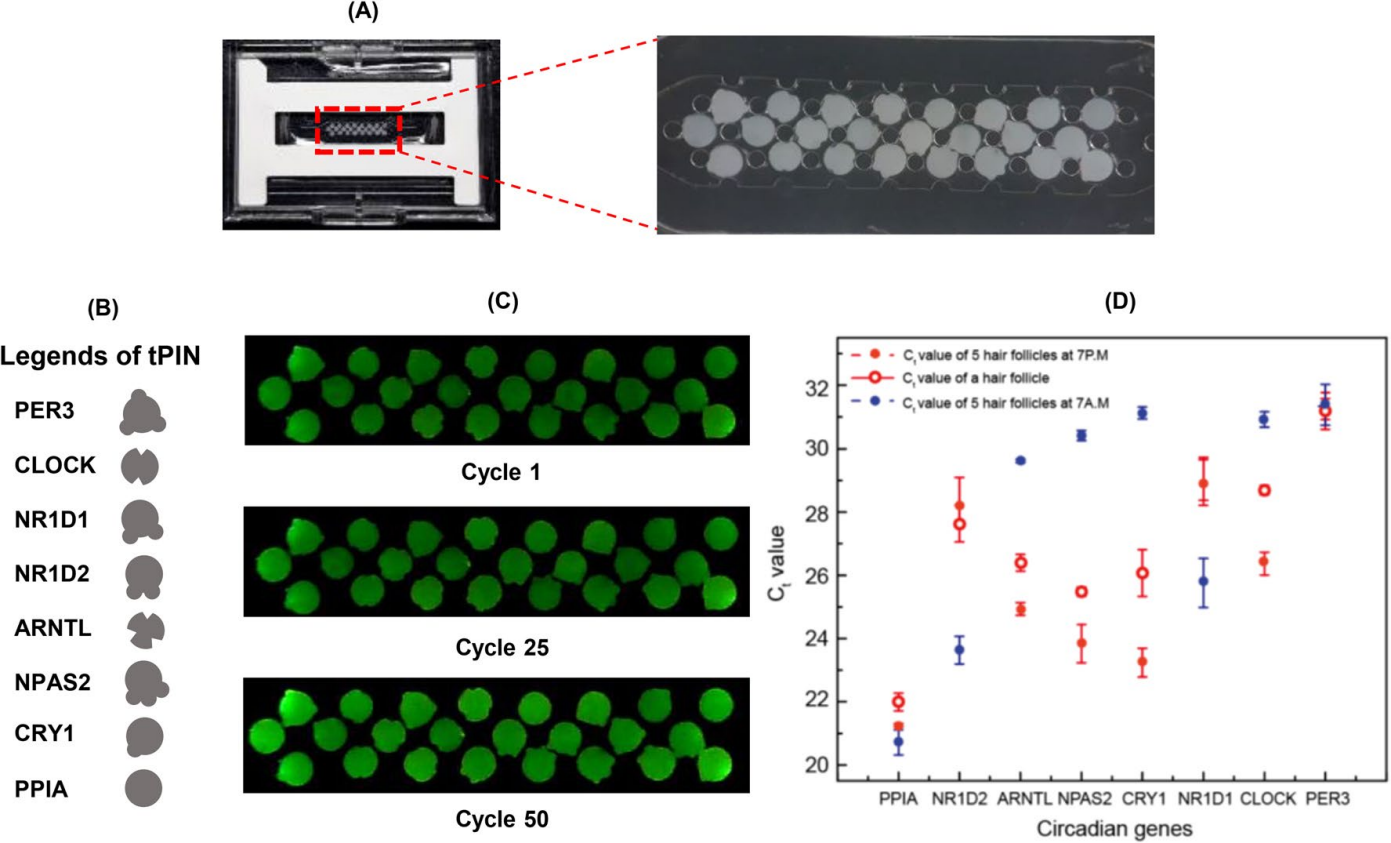
Snapshot from a single hair follicle. (**A**) Assembling three particles for 7 circadian markers and a 1 reference marker on to a plastic chip. (**B**) Specific slit codes for tPINto figure out the distinct circadian biomarkers. (**C**) Significant fluorescence was observed during the assay cycles of 25th, 50thcycle while absent in the 1stcycle of qPCRassay. 24 microparticleswere brightened separately. (**D**) 7 circadian markers and 1 reference marker showed individual Ctvalues simultaneously with standard deviation of 0.05 to 0.7. The assay was performed in triplicates (n = 3).

It is recommended to use 5–20 hair follicles for reliable measurement of multiple mRNAs due to variations across different subjects. Humans differ in the amount of RNA that can be extracted from each individual hair follicle because of experimental conditions, such as plucking follicles or not; these differences can affect the efficiency of RT-qPCR^23^. Although obtaining hair follicles is minimally invasive, it can still put stress and strain on a patient to remove 5 to 20 hairs at one time. Generally a smaller amount of sample is obtained with non-invasive interventions than is with invasive interventions. This is a practical factor to consider with regard to performing clinical studies of high consistency. We obtained a stable pattern of circadian gene expression from 5 hair follicles. Later, we examined a single hair follicle for multiple RNA analysis. Since a single follicle is known to have > 7 ng of total RNA^24^, we prepared a PCR chip of the particle array requiring a smaller amount of sample. We configured a 24-particle array that corresponded to eight genes (PER3, CLOCK, ARNTL, CRY1, NPAS2, NR1D1, NR1D2, and PPIA) in triplicate. (Fig. 6A) The expression levels of 8 targets were quantified using 8 uL of total RNA that was extracted from a single hair follicle. The slit codes were assigned to the sidewall of the particle to identify the particles with different primers. (Fig. 6B) 8uL of the total RNA was filled into the PCR chamber. The incubation for the pre-concentration was performed at 55° C for 5 min. As the reagents were introduced into the chamber, one-step RT-qPCR was carried out for 30 min. (Fig. 6C) The resultant C_t_ values for each gene are shown in Fig. 6D (red bold line). Approximately 50% of the total RNA from a single hair was enough to analyze 8 different genes with triplicate. The single hair was obtained at around 6 pm and this expression pattern was consistent with that of five hairs of 7 pm on the previous study. The profile was in contrast with that of 7 am. This consistency implies that the assay is reliable, even when using a single hair. Therefore, this tPIN assay is a powerful RNA analysis platform that can easily profile multiple RNAs from a small clinical sample.

## Discussion

We demonstrated the multiplexed one-step RT-qPCR platform using tens of tPIN microparticles. This platform is able to extensively profile mRNA from very limited samples. Using RNA capturing and pre-concentration, a single tPIN can quantify mRNA from just 200 pg of total RNA. We were also able to perform 24-plex mRNA analysis using a single hair follicle. This platform operates very simply and efficiently, especially given its reliance of a small amount of clinical sample. The 24 individual RT-qPCR was replaced with a single operation on a PCR chip without any loss of performance (including selectivity and sensitivity). As clinical targets expand for more precise diagnosis and evaluation, we believe our new platform will be advantageous over conventional assays. In addition to the circadian rhythm, many other disease-related transcripts can be monitored using non-invasive sampling and this versatile platform.

## Supplementary Information

Supplementary Information

## Abbreviations

cDNA: Complementary DNA
FBS: Fetal bovine serum
IVT: In vitro transcription
LOD: Limit of detection
MEM: Minimum Essential Media
NTC: No template control
oPIN: Original primer-incorporated network
PBS: Phosphate buffered saline
PDMS: Polydimethylsiloxane
PIN: Primer incorporated network
RT-qPCR: Quantitative Reverse Transcription-Polymerase Chain Reaction
tPIN: Thermo-responsive primer incorporated network
