# Supplementary material for: Highly sensitive and multiplexed one-step RT-qPCR for profiling genes involved in the circadian rhythm using microparticles
Source: Sci Rep. 2021 Mar 19;11:6463. doi: 10.1038/s41598-021-85728-y (PMC7979730; doi:10.1038/s41598-021-85728-y)
Supplement: Supplementary file 1 — Supplementary Information [file 41598_2021_85728_MOESM1_ESM.pdf]

Highly sensitive and multiplexed one-step RT-qPCR for profiling genes involved in the circadian rhythm using microparticles

Mi Yeon Kim1,2, Seungwon Jung1, Junsun Kim1,2, Heon Jeong Lee3, Seunghwa Jeong3, Sang Jun Sim2 and *Sang Kyung Kim1

1 Center for Molecular Recognition Research, Materials and Life Science Research Division, Korea Institute of Science and Technology(KIST), Seoul, KS013, Korea

2 Department of Chemical Biological Engineering, Korea University, Seoul, KS013, Korea

3 Department of Psychiatry and Chronobiology Institute, Korea University College of Medicine, Seoul, KS013, Korea

* Sang Kyung Kim
Email: sangk@kist.re.kr
Phone: +82-2-958-6794, Fax: +82-2-958-6910

(A)

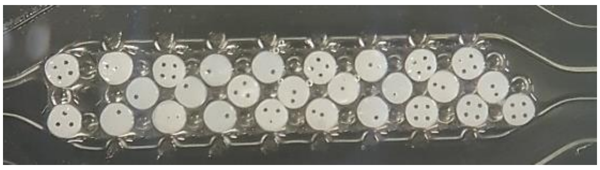

(B)

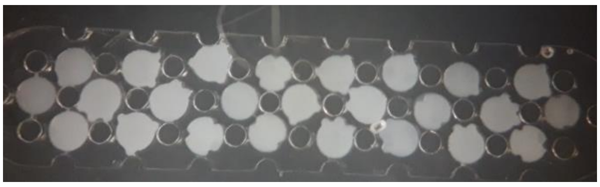

Figure S1. Pre-patterned PIN particles (A) Dot codes PIN particles (B) Slit codes PIN particles

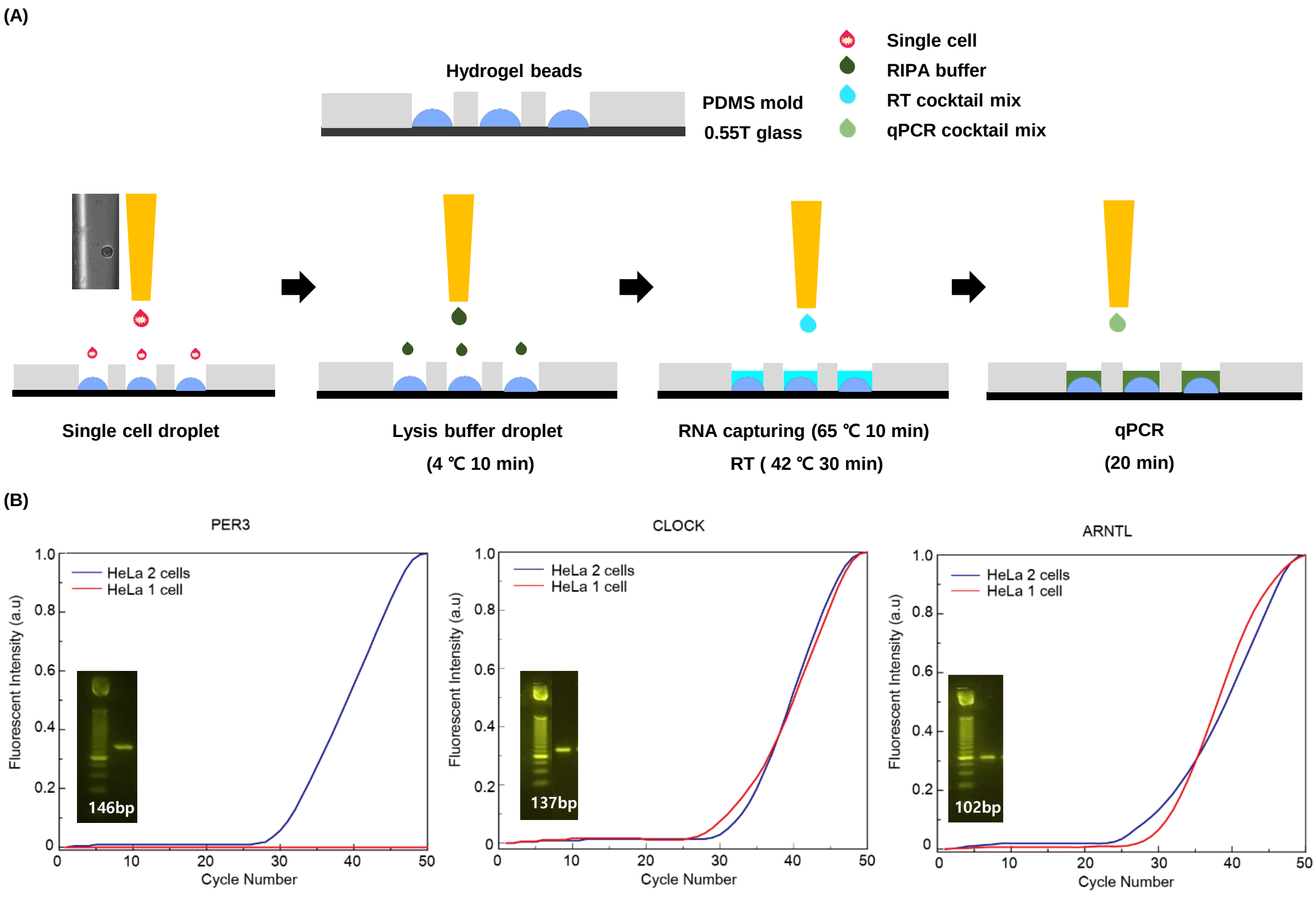

Figure S2. Single cell quantification by PIN based PCR. (A) Schematic overview of singe cell quantification by two-step RT-qPCR process (single cell to qPCR). (B) From five cells to single cell, qPCR were performed in the microparticle. PER3 gene was detected up to 2 cells whereas ARNTL and CLOCK genes was detected up to single cell level around 26-28 of C_t value. The electrophoresis data displayed only the target amplicon in the PIN particles after the qPCR process for HeLa cells.

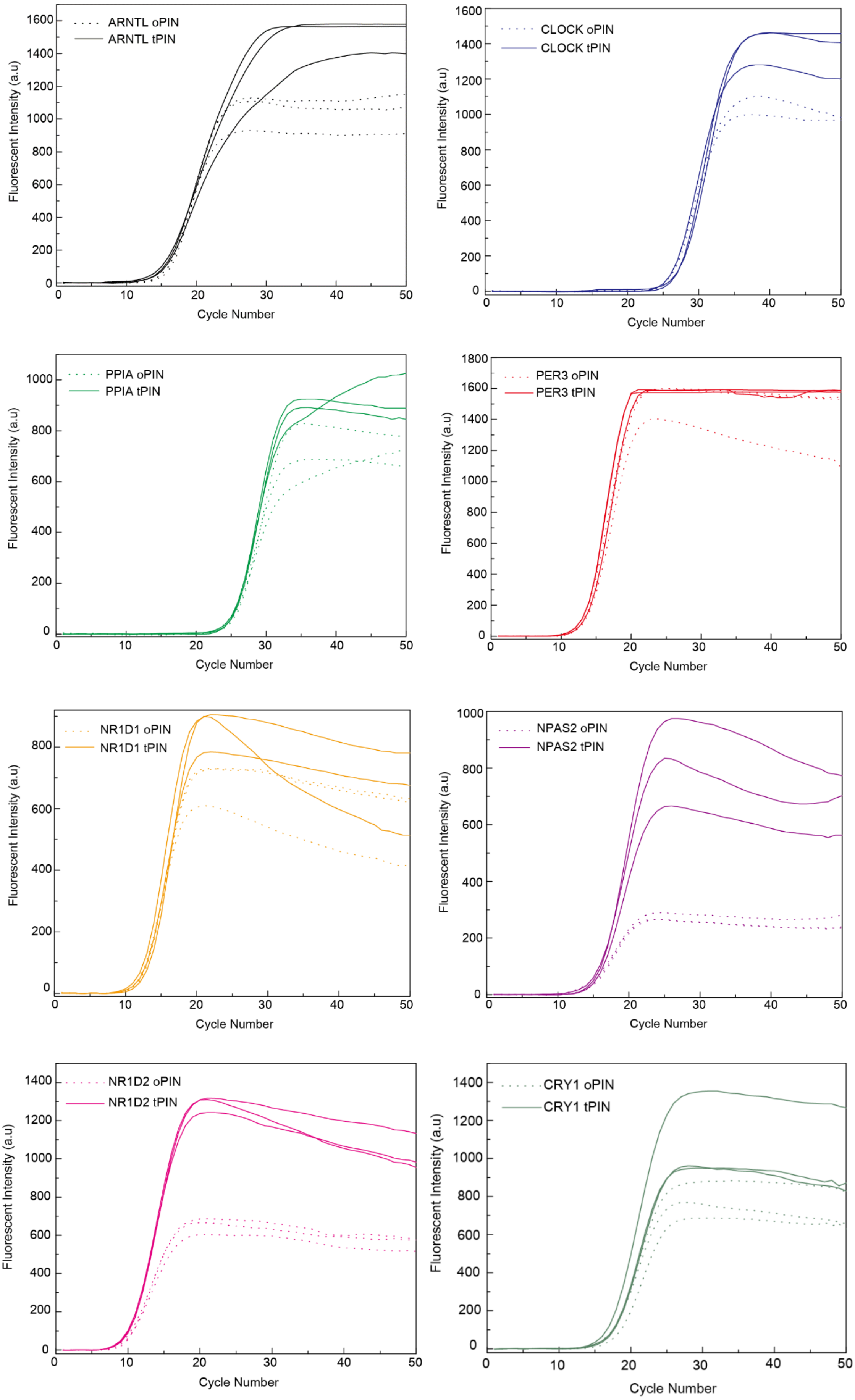

Figure S3. oPIN, tPIN validation of circadian rhythm markers. Different genes which target the circadian rhythms (*PER3, CLOCK, ARNTL, CRY1, NR1D1, NR1D2, NPAS2, PPIA*) were analyzed. qPCR was proceeded with the synthetic DNA of 10^7 copies / µl. The C_t value of tPIN and oPIN were similar. However, the fluorescent intensity in tPIN was higher than that in oPIN for each gene.

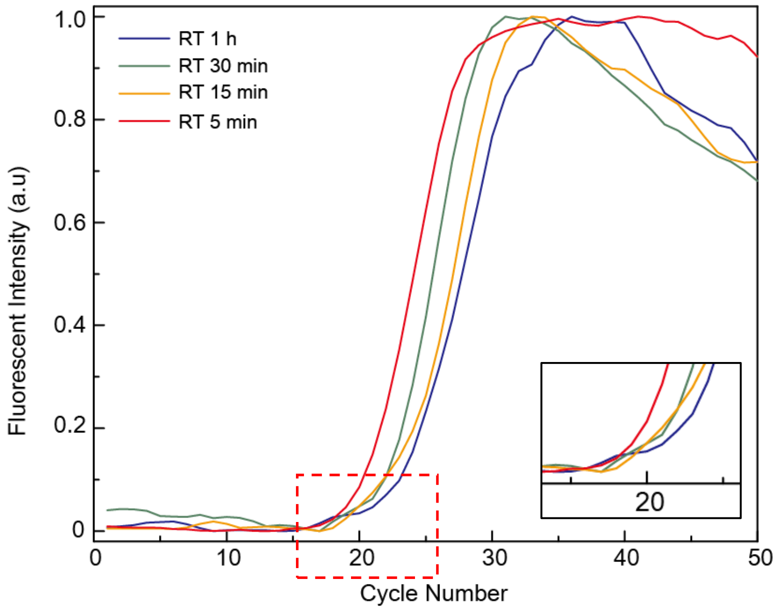

Figure S4. RT validation. The reverse transcription (RT) time was verified at 42 °C to confirm the highest RT efficiency. One-step RT-qPCR targeting of *CLOCK* IVT RNA was proceeded by changing the RT time from 5 min to 1 h at 42 °C. Four different time periods were checked and all of them possess the same C_t value. Therefore, we have chose 5 min as the sufficient time for effective rapid one-step RT-qPCR.

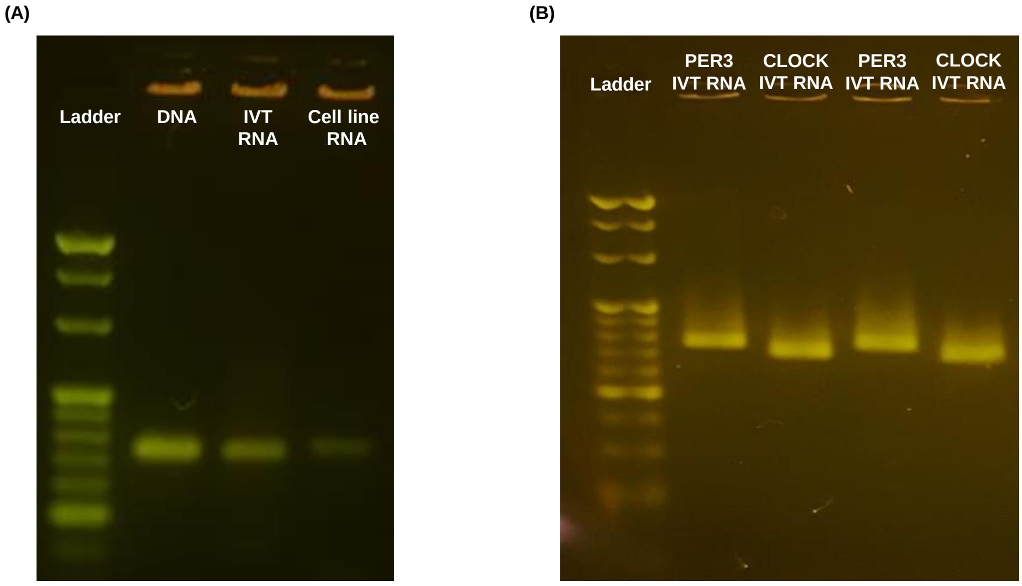

Figure S5. Gel electrophoresis of synthetic DNA, IVT (*In vitro* transcription) RNA, and cell line RNA. (A) One-step RT-qPCR gel electrophoresis data. All the three samples were utilized to perform PCR targeting for PER3, followed by electrophoresis where only the target band was amplified accurately even in high complex cell line RNA. (B) Gel electrophoresis of the synthesized IVT RNA for PER2 and CLOCK

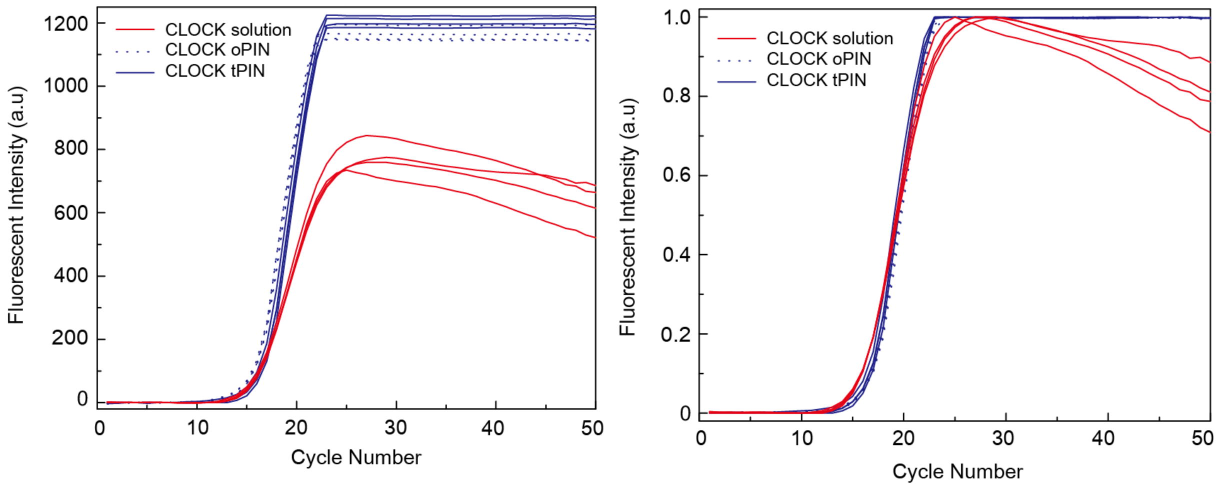

Figure S6. Comparison between the conventional and PIN (oPIN and tPIN) based one-step RT-qPCR. The C_t value of the PIN based RT-qPCR was delayed by 1.01 with standard deviation of 0.15 compared to that of the conventional solution RT-qPCR. C_t value of the particle qPCR (oPIN and tPIN) were similar.

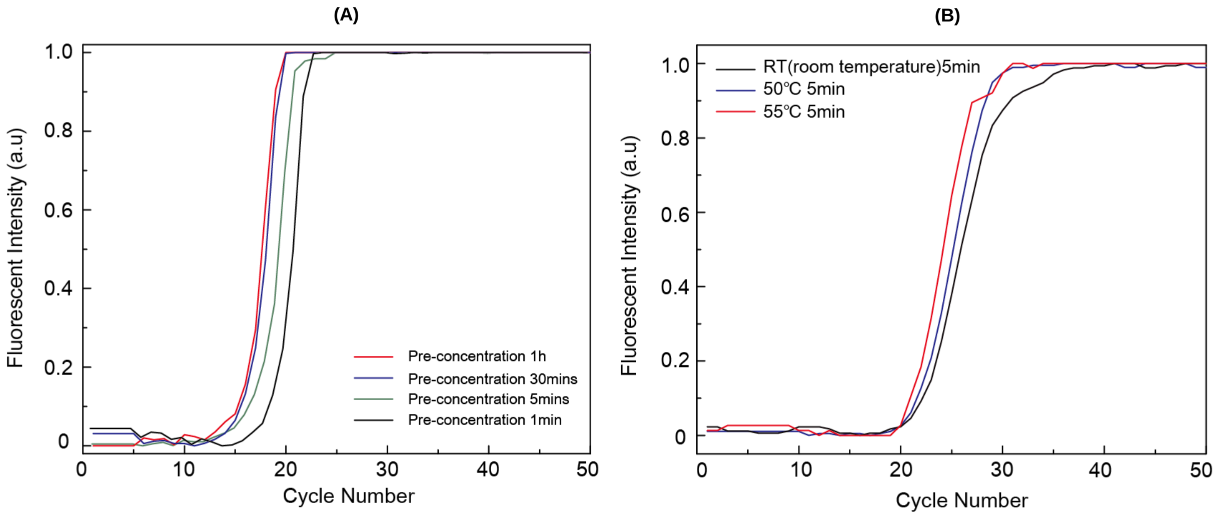

Figure S7. The condition of pre-concentration. (A) The various time conditions of pre-concentration. Except 1min, other expressions are similar. (B) The various temperature of pre-concentration.

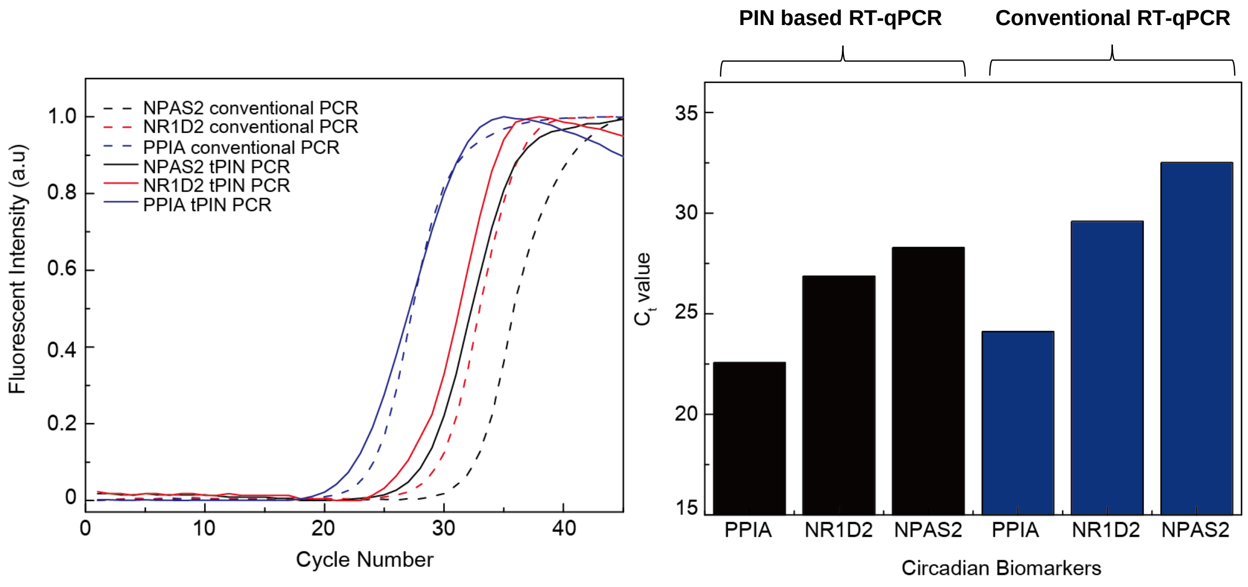

Figure S8. Comparison between conventional RT-PCR and PIN based RT-PCR. The fluctuations of expression level of each gene were similar. r. The left image showed the intensity of fluorescence in brightening particles per cycle in real time, and the right showed the Ct values read from the left graph to bars
